# Children’s Lived Experiences in Poverty in Hong Kong as a High-Income Asian Society

**DOI:** 10.3390/ijerph19106190

**Published:** 2022-05-19

**Authors:** Esther Yin-Nei Cho, Victor C. W. Wong

**Affiliations:** Department of Social Work, Hong Kong Baptist University, Kowloon Tong, Kowloon, Hong Kong; vicwong@hkbu.edu.hk

**Keywords:** child poverty, lived experiences, deprivation, social participation, coping strategies

## Abstract

Child poverty situated in different socioeconomic and environmental contexts has long been a central concern for practitioners, researchers, and policy makers. However, concerned research studies are predominantly adult-centric, confined to specific areas, or seldom found in Asian developed economies. Against the backdrop of this research gap, this study examines children’s experiences of poverty in relation to economic and material aspects, social relationships and participation, and psychological and emotional wellbeing, and their ways of coping with the effects of poverty. Using a purposive sampling method, a total of 40 children participants aged 8–14 living in or near poverty were recruited for an individual interview. The study showed that children experienced a range of deprivations in relation to falling short of the resources, opportunities, and activities that are commanded by average young persons. Limited living space also stands out as a more severe problem that is difficult to cope with. The various coping strategies include small spending savvy tactics, parental buffering, compensation, and mental coping. Proximity to schools and NGOs can help children in poverty to cope with problems caused by deprivations in different aspects. Implications for research studies and practice for working with children in or near poverty are discussed accordingly.

## 1. Introduction

Child poverty has long been a central concern for practitioners, researchers, and policy makers. Even in high-income countries, one in five children lives in relative income poverty, as measured by household income being lower than 60% of the national median [1]. The body of literature on child poverty is exemplified by several characteristics. First, child poverty research tends to be dominated by the use of quantitative approaches. These approaches have contributed to revealing a wide range of statistical evidence of child poverty in regard to its prevalence and trends [1], persistence [2], depth [3], risk factors [4], and consequences [5].

Second, the literature tends to be adult centric. In pertinence to the prevalence of quantitative investigation, children are often subsumed within families or households, and therefore the views of adults are emphasized [6]. Children are also presumed to be incapable of understanding and evaluating their life circumstances [7], thus resulting in parents speaking as proxies [8]. However, in the wake of the ratification of the United Nations Convention on the Rights of the Child [9], children not only have the rights to a standard of living adequate for physical, mental, spiritual, moral, and social development as stipulated in Article 27 of the UNCRC, but their rights to be heard are also acknowledged, as set out in Article 12. Moreover, the idea that children and young people should be regarded as social actors in their present lives has been recognized in research studies [7,8,9,10,11,12,13,14].

Third, research studies addressing the first two issues are especially scant in the high-income Asian context. The last two decades have seen a growing effort to conduct child-centric poverty studies by employing both qualitative and mixed methods approaches. However, the qualitative investigations that engage children’s perspectives are mostly found in Western developed countries [15,16,17,18], Sub-Saharan Africa [19,20], and other developing countries including those in Asia [21,22]. Limited research of this kind has been conducted in developed Asian societies such as Hong Kong. Hong Kong is one of the most affluent cities in the world; its GDP per capita was 46,324 USD in 2020 [23], and one in every 125 residents has a net worth of at least 5 million USD [24]. Nevertheless, the Hong Kong economy is also one of the developed economies with the highest income disparity, with a Gini coefficient of 0.553 in 2016 [25]. The poverty rate for the whole population was 23.6%, compared to 27% for the child population [26]. Most of the relevant research studies in Hong Kong focus on income deficit and deprivation as the measures, whilst studies on personal experience are sparse [27]. Ho and colleagues [28] compared the daily life experiences of children from low and high-income families, but the study only focused on health behavior, showing how lifestyles and eating habits mediated the effects of income disparity on the psychological wellbeing of children. Spires [29] studied youth perceptions on social mobility barriers, but the study itself was limited to participants recruited from a small geographical area.

Against the above backdrop, this study fills the gaps by conducting a study to understand children’s experience of poverty in Hong Kong. Children gave their perspectives on how poverty affected their lives and how they coped with the impact of poverty. The findings of this study not only provide insights into measures that may help improve children’s life conditions in Hong Kong, but the study also adds to the current literature of child poverty by taking into account the less heard perspectives of children from a high-income society in Asia. In the following, the literature relevant to child poverty study is concisely provided, which is followed by the Methods section. Then, the findings of this study and its implications are presented accordingly.

## 2. Literature and Findings on Children’s Experience of Poverty

With a recognition that children are social actors [7,8,9,10] and that they should be provided the opportunities to express their views [9], the literature has examined how children cope with the effects of poverty by taking into account the different aspects of their lives. Existing studies show that the experiences of poverty can be categorized into three major dimensions: economic and material deprivation, social relationships and social participation, and psychological and emotional wellbeing [30,31,32,33].

First, in some studies. children reported on the negative effects of limited income and material deprivation on their quality of living. They talked about unmet basic needs in terms of food, accommodation, clothing, and health care; limited childhood possessions in terms of toys; and inadequate schooling resources, such as school uniforms, information technology devices, and school trips [34,35,36,37,38]. For example, in a study of a group of children aged 9–13 sampled from Supplemental Nutrition Assistance Program-eligible households in Minnesota, U.S., it was reported that they consumed low-cost, high-sugar, or high-fat food more often than more expensive, healthier food, which was less available to them [35]. In another study, a group of Australian homeless children aged 6–12 from diverse ethnic backgrounds experienced a range of 3 to 11 changes of temporary accommodations, such as motels, sleeping rough or in cars, and caravan parks, which adversely affected their physical and emotional wellbeing [37].

Second, poverty has negative impacts on children’s social relationships and social participation. In some studies, children showed that they could not afford to invite friends over or go out with them to play [38,39,40,41]. For instance, in a study of children aged 10–17 in England, some children were unable to have sleepovers at home because their homes were too small or they could not go to see their friends due to a lack of transportation [40]. They felt the pressure of not having the right clothing, trainers, or accessories as status markers to keep up with their peers [16,42,43,44]. For some children, teasing or bullying could be experienced due to being unable to fit in with their peers [42,45]. It was shown in a study of children aged 6–16 from poor families in Netherlands that avoiding contact with peers could be a way of coping, such as by running away from school to avoid being bullied or by not disclosing their birthday to conceal the fact that they did not have enough money to hold birthday parties [46]. Children were also less likely to enjoy leisure or organized activities because they could not afford the costs of transportation and activities [15,16,41]. In a study of children in poverty in Sweden, some children said that being unable to afford the same things as their peers and being forced to skip activities led to stress and anxiety [15]. In other studies, some children would limit their leisure activities in their low-income neighborhood where they felt unsafe due to violence or other reasons [41,47,48].

Third, some research studies show that children’s psychological and emotional wellbeing can also be affected. Children felt fearful and disappointed when family income deteriorated. In a longitudinal study of low-income, lone-mother households in England, some children, who were aged 8–15, expressed fears of a return to their prior financial situations when their mothers were unemployed, as they could recall clearly how the negative effects of poverty had penetrated into their lives [49]. Even young children worried about their family circumstances and parents’ physical and emotional health [37,50]. In the study of a group of homeless children in Australia, some children were often concerned about the health and mood of their parents, and such concern about their parents was often associated with a child assuming responsibility for protecting their parents [37]. Some studies show that children could feel embarrassed or stereotyped by others in school and in the community [42,43,45,51]. For example, in a study of homeless children aged 8–13 in Southern California, U.S., some children felt ashamed when other children knew they were poor as they were offered free backpacks, coats, or school trips [42]. Children’s expectations for the future were mixed. Some children believed they would have good educational and career prospects, while others were not optimistic [38]. Children’s aspirations tended to be modest when they needed to assume family responsibilities early in life, when they got used to a modest lifestyle, or when they were aware of their limited opportunities due to socio-economic or other environmental barriers [40,41,45,52].

Drawing on the ideas that children are capable of acting upon and interacting with their social worlds, that children should be given the rights to be heard, and that the impacts of poverty are multidimensional, this study aims to understand children’s experience of poverty in Hong Kong by listening to their perspectives on how poverty affects their lives in different aspects and how they cope with those effects of poverty. Based on existing literature, the research questions are as follows:

(i) What are children’s experiences of poverty in the following aspects:
  - economic and material deprivation, including basic needs (food, clothing, housing, and health care), childhood possessions (toys), and schooling resources?
  - social relationships and social participation, including peer relationship, family relationship, and participation in extra-curricular activities?
  - psychological and emotional wellbeing, including feelings about family circumstances, feelings about school lives, and aspirations?
(ii) How do children cope with the effects of poverty?

This investigation is concerned with the study of the socioeconomic, relational, and environmental factors leading to the lived experiences and wellbeing of children in or near poverty. It addresses the important topic of child poverty and its alleviation in relation to promoting the wellbeing of children with reference to a wide spectrum of environmental factors including community facilities, school readiness, and use of other relational and health resources.

## 3. Methods

### 3.1. Approach

This study adopted a qualitative approach, using individual in-depth interviewing to gather children’s perspectives on how poverty affected their lives and how they coped with it. The data acquired from a qualitative investigation may provide a deeper understanding about the richness and complexity of human behavior. With the aid of semi-structured questions, the children are provided the opportunities to express in their own words how they felt about their daily life experiences. The use of individual interviews is appropriate, as it is agreed that those who are as young as seven are capable of providing meaningful and insightful information [53,54]. Existing research studies on children’s poverty experience have also been conducted with children as young as six [37,55].

### 3.2. Participants and Data Collection

Using a purposive sampling method, a total of 40 children aged 8–14 living in or near poverty were recruited via non-governmental organizations (NGOs) to participate in individual interviews. Children who live near poverty were also included because their families may struggle to make ends meet on a daily basis even if they are not officially below the poverty line. In Hong Kong, the official poverty line is defined as 50% of the median monthly household income adjusted by household size before policy intervention. However, there is no official definition of near poverty. To cover different children who live in poor economic conditions, whether under or near the poverty line, the inclusion of children for the study was determined by their financial assistance status and other relevant socioeconomic backgrounds. Children of different backgrounds were included if they came from households participating in the Comprehensive Social Security Assistance Scheme (CSSA), Working Family Allowance Scheme (WFA), School Textbook Assistance Scheme (TA) with full grant, “N-nothings”, and new immigrants, which are introduced regarding their basic features below.

The CSSA Scheme recipients are typically considered to be individuals who live under the poverty line. The scheme provides assistance to people who cannot support themselves financially by bringing their income up to a prescribed level to meet their basic needs [56]. The WFA scheme provides an allowance to households if they meet certain working hour requirements, along with income and asset limits. This allowance can be offered at full, three-quarter, or half rates. The upper limits of monthly household income for full, three-quarter, and half rate allowances are 50%, 50–60%, and 60–70%, respectively, of the median monthly household income [57], indicating that those who receive the full rate are under the poverty line, while three-quarter and half rates are marginally above the poverty line. The School TA scheme assists primary and secondary students who are in financial need, for covering the costs of textbooks and miscellaneous school-related expenses [58]. The level of assistance, offering at full or half grants, is assessed by using an Adjusted Family Income (AFI) mechanism that takes into account the gross annual income of the family and family size. A full grant indicates a greater financial need. The category of “N-nothings” refers to low-income households who do not benefit from any government welfare due to different reasons, such as those slightly exceeding the income or asset limits for CSSA, public housing, or WFA. These households, who fall through the social safety net, live in private housing in poor housing and environmental conditions and struggle to get by. New immigrants refer to those who immigrated to Hong Kong from mainland China in the past five years. They tend to have lower household incomes due to a generally lower educational level and greater engagement in low-skilled jobs [59]. They are more likely to be prone to poverty—their poverty rate (30.1%) was double the poverty rate for all households (14.7%) in 2016.

Prior ethical approval for the study was granted by the Research Ethics Committee of the university that the authors are affiliated with. Informed consent from both the children and their parents was obtained before the children participated in the study. The purpose of the study and the principles of confidentiality and voluntary participation, which emphasized the right to discontinue the interview at any time, were communicated to the participants before the interviews started. All the children were recruited through NGOs that have connections with low-income families. Each of the interviews took place in the NGO venue, which was familiar and comfortable to each participant, and generally lasted about 75 min.

As the interviews happened to take place largely during the time when the pandemic situation was relatively under control between the fourth and fifth waves (summer 2021) of the COVID pandemic, the study could capture some of the impacts of the pandemic on the children, even though its focus was not on the pandemic.

Table 1 shows the background characteristics of the children participants aged 8–14 who are all anonymized with a gender-specific pseudonym, studying at the level of Primary 2 to Secondary 1. Regarding gender, 22 were males while 18 were females. The majority of children had both parents at home. Most of the children received a full grant from the School TA Scheme, while others were spread across different types of financial assistance or backgrounds.

### 3.3. Data Analysis

Thematic analysis was adopted to identify, analyze, and report meaningful themes across the interview data [60]. After familiarization with the data, the meaningful elements in the data were identified to generate initial codes. Within each of the aspects in children’s experience of poverty, the different codes were categorized into potential themes, and the relevant coded data were collated within the identified themes. Attention was also given to the codes relating to children from different financial assistance and socioeconomic backgrounds so that their experiences could be well included in the themes. It is also possible to discern potential patterns of differences in the effects of poverty across different backgrounds. The potential themes were then reviewed and modified by making sure data within themes were coherent, while themes were distinct from one another. After all refinements, the themes were named to capture the essence of what each theme was about. The researchers held on-going discussions on the analysis of data. The themes were refined and organized until consensus was reached.

## 4. Results

### 4.1. Economic and Material Deprivation

#### 4.1.1. Basic Needs

##### Being Food Secure without Going Hungry

The children appeared to be food secure as food was available to them and they did not have to worry about getting hungry. They typically had three meals a day, and they felt that they had a balanced diet. Most of the children reported that they had breakfast, lunch, and dinner every day, though some of them sometimes skipped breakfast mainly due to getting up late on school holidays or during school suspension amidst the pandemic. Not many children had afternoon snacks or had more than three meals. Most of the children expressed that they had sufficient food to eat, even though some children had to juggle their resources to access food. For example, a child’s family was provided with fish from an NGO three times a month. As she wanted more fish in her diet, she showed how her family stretched their budget and got bargains in the supermarket or wet market near its closing time for inexpensive fish or other food.

*We’ll buy fish if it’s cheap, about HK$10 or $20 for one piece. …If someone gives us supermarket vouchers, we’ll buy them from the supermarket …and we’ve bought a few frozen fish for only $30 before! Frozen fish is better …because the fresh ones are raised in the pond, not in the ocean. …We go to* *the market around the time it closes. …There would be 3 or 4 portions of food for just $10. …It’s at about 6 to 7 o’ clock.*

(Eleanor, P3, CSSA)

Regarding nutrition, most of the children felt that their diets were balanced. They said that their meals at home usually included meat, vegetables, carbohydrates (bread, rice, or noodles), and sometimes soup. Nevertheless, the meals of some children may not have been that balanced, as some said that they often had processed food such as luncheon meat and mustard tuber, which contain high levels of sodium. A child described his usual diet without meat:

*In the morning, I have some crackers and a glass of milk. …These are provided by an NGO. … Sometimes, people might give us flour, and we would make something like Chinese pancakes. …When we are given macaroni, we’ll add some vegetables as a meal. [For dinner] …we may have noodles with vegetables, such as carrots or tomatoes. …During weekends, we cook one or two veggie dishes. My mom and I would each have a bowl of rice. …Sometimes the NGO gives us meat. …We would have meat once a month. … [During the week], we usually eat rice and vegetables.*

(Brandon, P3, WFA)

For children in this kind of situation, school lunch can become an important source of protein. Primary school children usually have lunches at school. Those who come from low-income families are provided with free lunches. Such an important source of nutrition for low-income children may have been interrupted by school suspension or the cancellation of school lunch during the pandemic.

##### Being Deprived of Socially or Culturally Desirable Food Practices

In addition to satisfying physiological needs, food and eating also has social and cultural significance in communicating information in terms of social status, ethnicity, and wealth [61]. While the children have enough food for consumption, they may be falling short of food or eating practice that is socially or culturally desirable.

Dining out is commonly seen as a social activity for enjoyment. However, the majority of children said that they had meals prepared at home in most situations, except in the case of having free lunch at school or takeaway food, even before the pandemic. For most of the children in this study, dining out was only occasional, usually once a few months or a few times in a year. Some children might also share a single set meal with their parents when eating at fast food restaurants. When dining out, the popular choices were fast food, budget Western food, or sushi. A female participant was delighted to describe her favorite sushi-go-round:

*It costs only eight dollars [about US$1] for a dish [of sushi] …and I had seven dishes! …I ate even more than my brother. …We went there once a year but this year I went twice, as I went with my cousin last time.*

(Jenny, P3, new immigrant)

While the children enjoyed eating out, they were modest in asking for more. Some said that they did not strongly desire fast food as it was expensive or their parents told them it was unhealthy.

Seafood is a popular delicacy in Chinese food culture, but it is infrequently found in their meals, except for small, inexpensive fishes. As seafood is relatively expensive compared to other daily foods, some children could only eat seafood on special occasions such as birthdays, when it was provided by NGOs, or when the parent brought home leftover shrimps and crabs from the workplace. In an extreme example, a male participant (Brandon, P3, WFA) had never eaten certain popular seafoods, such as clams, crabs, and lobsters.

##### Limited Living Space

The children mainly resided in two types of housing: subdivided flats in the private market and public rental housing estates. Subdivided flats, or “butchered rooms”, are residential flats further partitioned into two or more separate units to accommodate more people at lower rent prices than the whole flat would have. Public rental housing estates refer to housing programs of the government for low-income residents, who are unable to afford private housing. The median per capita floor area of subdivided flats was 5.3 sq m in 2016 compared to 11.5 sq m for public rental housing and 18 sq m for private residential flats [62].

The living space was usually cramped in subdivided flats. In worst-case scenarios, there was no bedroom, and the family members needed to share beds. A boy said that he slept on the upper bed of a bunk bed while his parents and young brother all slept on the lower bed (Perry, P5, TA). The children themselves also felt the living units were small and crowded with too much miscellany.

*There is no partition at my home. We don’t divide it into rooms …everything is in one place.*

(Gerald, P3, new immigrant)

*My mom put a cupboard by the door. It makes it impossible for me to close the door.*

(Ian, P3, new immigrant)

In public rental housing, there is relatively more space with bedrooms. A child (Isabel, P6, new immigrant) compared her current home with her previous ones. The best home was the current flat with three bedrooms in a public housing estate. The second best was a unit in a walk-up tenement building in an old residential area that was still relatively spacious. The worst was a subdivided flat in one of the most densely populated districts. The flat was so small that it was difficult to even turn around.

Overall, the home environment of the children was small, crowded, and even noisy. A child shared that he wanted to retain moments of quietness by having a pair of earphones for use.

*I want to listen to music quietly …because my home is noisy. …I would do it under my blanket. It’s so quiet, as if the world was so quiet. …As I have no earphones, this is the only solution.* (Nick, P5, TA)

##### Minimal Set up at Home

Many children remarked that most of their furniture was old and the set up at home was minimal. In certain situations, there was no stove for cooking, and so a basic electric rice cooker was used to do all types of cooking. When asked about their wish list for home, most of the children wanted more space for themselves, such as having their own bedroom as well as various basic home items, including a desk, a bookshelf, a printer, an oven, a sofa, a mirror, a washer, a camera, or a vacuum cleaner. Other than these essentials, most of them were able to get along with their status quo.

Learning from her parents, another child (Bridget, P4, WFA) emphasized that it was necessary to distinguish between wants and needs. Her family had to buy a washing machine and refrigerator to satisfy basic needs, but the model kits her parents did not buy for her were just wants.

##### Sufficient in Quantity but Not Necessarily in Quality of Clothing

Most of the children found clothing for both warm and cold seasons sufficient. School uniforms were also sufficient, as they typically had two interchangeable sets. Having school uniforms could help reduce the need for extra daily clothing on school day. Some even wore their school uniforms until they went to bed. In general, they got a mix of new clothes and hand-me-downs from their elder siblings or cousins, friends, or NGOs. A female participant expressed that her clothing needs were given priority over her mother’s.

*My mom wears my old shoes and outfits …so she doesn’t need to spend on her clothing. …Just like she lets me eat more of her food while she eats less.*

(Flora, S1, CSSA)

However, there is disparity in understanding the sufficiency of clothing. Some children said that they had a great deal of clothing, such as more than 40 sets of outfits for both summer and winter, while some had around 10. In addition, the clothing may be sufficient in terms of quantity but not necessarily in quality, as there could be a large number of insufficient clothes.

*I have a lot of clothes, but they are not worn …they do not fit my size.*

(Rex, P4, TA)

*I have 20 to 30 tees …and around 20 pairs of pants. …But a lot of them were worn in kindergarten years and are put in the bottom of the shelf. …The tees I often wear are fewer than 10.*

(Kate, P5, CSSA)

Most of the children felt that their clothing was not much different from other children, though some said that they noticed the “prettier” and “more fashionable” outfits of their peers (Flora, S1, CSSA). Generally, the children did not show a great desire for more clothing for different reasons, such as they had sufficient clothing or it was expensive. Those who had relatively fewer clothes also said that only a few clothes were worn either way, so it did not bother them.

Nevertheless, this did not mean that the children did not care about clothing. They understood that decent clothing was important to their social lives. For example, a female participant (Olivia, P5, TA) shared that wearing sleepwear to her classmate’s home was improper, even though they lived in the same housing estate. She would also dress better when going to the town center.

##### Heavy Reliance on Informal Health Care

Apart from minor conditions such as short-sightedness, flu, and cold, the children generally felt healthy and did not have major health complaints. In the case of sickness, both formal and informal approaches to health care were adopted, but the latter was more frequently practiced.

Formal health care was particularly used when the children required more urgent medical attention, such as contracting measles or a fever. When using formal health care, the children were more likely to visit low-cost government clinics or public hospitals than private doctors. With regard to more expensive private health care, the children of the family tended to be given priority in seeing private doctors. A child said that she would be taken to visit private doctors when feeling unwell, but her mother would not (Eleanor, P3, CSSA). Another child said that whether or not he could see private doctors would depend on his family’s financial situation at the time (Todd, P5, TA).

Informal health care among the children refers to taking over-the-counter medicines or employing layman methods or home remedies. The over-the-counter medicines could be store-bought or sent by relatives from mainland China.

*We have this good medicine from mainland China. …You will feel well after taking it for a few days. I take it whenever I have a cough, fever, or flu. …We ask our relatives to send us more if it’s running out.*

(Queenie, P3, N-nothings)

Other layman methods included taking homemade herbal medicine, “sweating out” a fever, jogging, and simply taking a rest.

*There’s no need to take medicine. I’ll get well in a few days …just waiting to get well.*

(Kate, P5, CSSA)

*My parents asked me to take a flu formula drink and then run for a few laps in the basketball court. …When I have a fever, I’ll tuck in the blanket. I’ll get well after sweating.*

(Olivia, P5, TA)

Some children were aware of the importance of maintaining good health, and they said that they would do physical exercise so as not to get sick. A child said that *“I’ll go to my friends’ places and jog with them, play madly, and then I’ll sweat more. This will make me healthier!”* (Olivia, P5, TA)

#### 4.1.2. Childhood Possession

##### Lack of Popularity of “Traditional Toys”

The engagement in conventional styles of toys, such as dolls, stuffed toys, cars, craft kits, and board games, is commonly thought to be beneficial to children’s cognitive and emotional development, in addition to its functioning as a means to pass the time. A small proportion of children in the study reported that they had a good quantity of toys, including LEGO, teddy bears, spinning top, dolls, puzzles, and ping pong. They got the toys from their parents or NGOs as gifts, hand-me-downs from their relatives or friends, DIY items, or prizes from Pachinko ball games. These children thought that they had sufficient toys or toys that were comparable to those of other children.

However, the majority of children did not have many toys. The first major reason given by the children for not having or playing with toys was that they felt they were too old for toys. As a female participant commented, *“…dolls are too childish. I have already grown up!”* (Claire, P4, CSSA). Space was another reason for the possession of few toys.

*It’s not easy each time I sleep. …As I don’t want to crush them, I have to sleep like this [gesture indicating the narrow width of the bed space]. …The bed is also filled with lots of stuff, like my school bag and my dad’s stuff. …I don’t know where I could put my dolls!*

(Giselle, P3, CSSA)

These children appeared to be indifferent even when noticing that they had fewer or almost no toys compared to other children. For example, a child said, “*I don’t feel much …because I don’t like playing with those toys …so I don’t mind.*” (Bridget, P4, WFA)

##### Fondness for “Modern Toys”

While the majority of children said that they did not strongly favor traditional toys, most of them preferred or viewed video games or smartphones as substitutes. When asked about their toys, some would refer to playing video games, such as *“…I play with PUBG …and Roblox …all the time.”* (Jenny, P3, new immigrant). Even without video games, many children made use of their smartphones for fun, including mobile games and social media platforms. The children were more aware that they were different from other children in respect to the possession of video game consoles or gaming systems. Some could see that all their classmates had video games but not them (Nick, P5, TA). Some of them would like to have Nintendo Switches or Sony PS5s.

#### 4.1.3. Schooling Resources

##### Lack of Non-Information Technology Resources

Schooling resources can be divided into non-information technology and information technology resources. Some of the home items the children expressed they needed were non-information technology resources, including a study desk and a bookshelf, which are difficult to fit in their tiny living space. For example, a child complained, *“I want to have a desk for doing homework, because I really don’t want to do my work on the small dining table every day.”* (Queenie, P3, N-nothings).

##### Lack of A Printer

Printers are a major information technology device for educational purposes. As some of the participant’s homework required submissions of printouts, a lack of a printer at home was a barrier to doing schoolwork. They sought assistance from different people, such as school personnel, NGO staff, or friends, but they found it troublesome.

*If I do homework at night and cannot ask people to print it out …I’ll copy it out by hand. …Yes, I copy it out by hand for my homework.*

(Eleanor, P3, CSSA)

*I have to ask the school social worker to help me with printing. …It’s about once or twice a week, not that often. …Still, I want to do it at home so as not to keep bothering other people. …It’s bothersome to me and the others who help me.*

(Claire, P4, CSSA)

##### General Accessibility of Other Digital Devices

The COVID-19 pandemic has largely upset educational practices both worldwide and locally. It has given prominence to the importance of digital devices as schooling resources and poses challenges to children in poverty in online learning. Fortunately, the children in this study generally had access, in varying degrees, to digital devices for online learning. Some children owned old computers, tablets, or smartphones at home, whereas some children were given used notebooks or tablets by NGOs. When compared with using desktop computers, some of the children preferred using smartphones to avoid capturing and showing their home surroundings when the computer camera was on.

*I use a cell phone to attend Zoom class. …I have a computer but it’s slow. It’s placed next to my bed, so you can see a lot of old stuff …and personal things on the bed. …It’s embarrassing. I don’t want people to see them.*

(Daniella, P6, WFA)

In general, children reported that the Wi-Fi connection speed at home was acceptable, though a number of children found that their connection lagged at times. It was also said that earphones were important gadgets given their tiny home environment. They wanted working earphones so that they would not be disturbed by and cause a nuisance to family members during class.

*Sometimes the earphones are out of order. …I’m worrying about it …because sometimes my family members are around …I don’t want to disturb them.*

(Claire, P4, CSSA)

### 4.2. Social Relationship and Social Participation

#### 4.2.1. Peer Relationship

##### School as A Major Setting for Friendship

Most of the children stated that school was an important place to enjoy interactions with friends. During school suspension due to the pandemic, maintaining interaction with their friends from school became harder. However, it was more difficult for certain poor children to maintain their peer relationships as some of them did not have their own smartphones. Some of the children hung out with friends via online games or in the nearby park. A child said, “*Now my best friends are those who play online together …it’s some online games, not those kinds of games that are found in school.*” (Daniella, P6, WFA). Home environment may be a factor that affects children’s social relationships. Many children said that they had never invited their friends over to their homes due to the lack of living space.

##### Limited Opportunity to Celebrate Special Occasions with Friends

The celebration of special occasions is an opportunity for individuals to spend time with friends. Birthday parties, for example, may be particularly important occasions for children, where they may feel special and emotionally connected to their friends. However, most of the children in this study did not hold birthday parties, and only a small number of them had participated in others’ birthday parties. For some children, birthday parties were not a common practice within their peer circles of similar backgrounds. For other children, preparing birthday presents was a disincentive for them to attend birthday parties.

*I have never been to others’ birthday parties. …I don’t want to …because it costs money to bring birthday gifts.*

(Wallace, P5, N-nothings)

It seemed to be more frequent for them to celebrate their birthdays with their family by eating special meals or cakes at home.

*If it’s my birthday, my dad would bring home five big macs! …Because we have five people.*

(Todd, P5, TA)

#### 4.2.2. Family Relationship

##### Family Interactions Depending on Parental Work

The children generally enjoyed their relationships with their parents, despite a few children saying that they rarely talked to their family members. A child enjoyed her relationship and chats with her parents very much, even though her father remained in mainland China and her mother was busy at work.

*We have good relationships and we chat often. …I do a WeChat video with dad once every two weeks because he is busy. …Mom is also busy …she works from 8 am to 5 pm …and sometimes until 8:30 pm.*

(Queenie, P3, N-nothings)

Parental work hours have a crucial influence on the communication between parents and children. Their communication and interaction could be limited, especially if the parents had long and irregular work hours.

*I talk to my dad only when he takes his day off. …He works from 1 pm to midnight …I am already sleeping when he comes back.*

(Bridget, P4, WFA)

*Mom has to go to the bakery to wash dishes after she finishes her first job. …So, she asked us to go play basketball, look at toys at toyshops, play with Pachinko games at the arcade … or just stay at home.*

(Gerald, P3, new immigrant)

##### From Simple Family Leisure to Cross-Border Trips

Some of the children reported that their parents would spend time with them during weekends or holidays if they were available. Most of them enjoyed simple leisure activities, such as going to the park, walking along the pier, hanging out in shopping malls, or playing poker or other board games at home. During long holidays, such as Chinese New Year or summer break, it was common for the children and their families to go to mainland China where they could enjoy spacious accommodation and delicious food at lower costs, especially when they had family connections over there. However, affordable cross-border trips have been disrupted by the pandemic.

*The food is much better than in Hong Kong. …There is so much going on …like there are many toys over there.*

(Perry, P5, TA)

*When I went to my hometown, my grannies would buy me whatever I wanted to eat. …I always said I wanted candies. …It’s only $1 per pack!*

(Jenny, P3, new immigrant)

#### 4.2.3. Extra-Curricular Activities

##### Generally High Participation in Free-of-Charge Extra-Curricular Activities

Most of the children welcomed and participated in a variety of extra-curricular activities, especially when the activities were offered for free by NGOs, schools, or churches. The types of activities included computer programming, choir singing, scouts, practicing magic tricks, swimming, piano playing, and dancing. However, the activities were largely interrupted during the pandemic. In a particular example, a female participant (Eleanor, P3, CSSA) expressed that she found her activities a bit excessive prior to her school’s closure. She had piano lessons on Mondays; Cambridge English lessons on Tuesdays; phonics on Wednesdays; dance classes on Mondays, Wednesdays, and Fridays; English again on Saturday mornings; and lessons for liuqin (a Chinese string instrument) on Saturday afternoons.

Cost is a crucial determinant in deciding whether the children would participate in extra-curricular activities. They tended to participate if it was free of charge or offered at affordable prices.

*If the free quota is running out, the school would draw lots and there might be chances that I cannot join.*

(Derrick, S1, TA)

*I won’t join if they are charged because I want to save money …I want to save my mom’s money.*

(Nick, P5, TA)

### 4.3. Psychological and Emotional Wellbeing

#### 4.3.1. Feelings about Family Circumstances

##### Emotional Wellbeing Affected by Perceived Family Stability

Children’s perceptions of family stability appear to influence their emotional wellbeing. Some children expressed their satisfaction when they experienced harmonious family relationships and a stable economic condition for the family.

*It’s really good to have such a perfect family. …We have food and shelter …a place to sleep. It’s better than the beggars sleeping on the streets.*

(Claire, P4, CSSA)

However, some children noticed the unstable financial condition of their families, and this became a source of worries. A child was concerned with poverty, as his father had been unemployed for two years and was failing to get a job (Caleb, P3, TA). Some others worried about the financial stress of their family, even though their parents were employed.

*I’m afraid our home expenses will become higher and higher, because just the utility bills already cost about a few thousand dollars a month …I worry about it every day.*

(Rex, P4, TA)

*My mom needs to pay insurance every year. It’s expensive. …There is a textbook fee for primary 6. …It’s higher than what we paid for the year before. …In the past, we paid on a term basis, but now we pay for the whole year. …We worry about [the economic condition] once a month. We get the salary, and then we’ll need to see how it goes.*

(Perry, P5, TA)

#### 4.3.2. Feelings about School Lives

##### Mostly Satisfied with School Lives

The children generally expressed satisfaction with their school lives. They enjoyed positive relationships with friends and teachers. A female participant said, *“I feel happy …because I have many good friends …and teachers are also kind. …We chat and laugh together.”* (Natalie, P6, TA). They also looked forward to hanging out with friends during recess and lunch hour as *“this is the most joyful time”* (Zack, P3, N-nothings). Those who took up specific duties in school also felt satisfied as they enjoyed a sense of recognition. A male participant shared his joy of becoming a school prefect: *“Yes I’m happy …I’m even becoming a school prefect! …I have passed a screening test. …The teacher asked how one would handle unexpected incidents, such as what to do when students feel sick. …Of course, I said seeking help from the teachers.”* (Ian, P3, new immigrant).

However, some children might feel less satisfied with school lives when they found their teachers too authoritarian in their teaching style or when they felt bored with learning.

*The teacher scolds us all the time. It’s like they’re scolding the whole class when the teacher is scolding only one of the students. I feel like I am being scolded.*

(Claire, P4, CSSA)

*Just nothing. You just stay in your seat. There’s nothing special about going to the school.*

(Queenie, P3, N-nothings)

In addition, in the pandemic, the implementation of social distancing measures and the cancellation of recess and lunch time undermined their satisfaction. A child said that she felt unhappy going to school: *“I cannot leave my own spot. I am not allowed to talk to my friends!”* (Giselle, P3, CSSA).

#### 4.3.3. Aspirations

##### Different Levels of Aspirations

The children showed diverse aspirations for the future, including low, moderate, and high levels of aspirations. Their aspirations reflected a richness in extrinsic and intrinsic types of short-term and long-term orientations.

*Having low aspirations*. Low aspirations may refer to limited desires to set goals for achievement. A number of children appeared to not think too much about their future yet. It was possible that some children were less ready to set goals as they may have had varying paces of development, or they may have lacked stimulation to inspire hopes. When asked about who they wanted to be or whether they had any dreams, some said, *“I have never thought about this matter”* (Aaron, P5, WFA) or *“I don’t know yet”* (Amelia, P4, CSSA). Some were uncertain about what they wanted and responded that they might simply need a job to maintain their living just like other ordinary people did (Caleb, P3, TA).

*Having moderate aspirations.* Aspirations appear to be moderate when goals are set, even though they are relatively short-term or smaller. Some children responded with desires to fulfil certain concrete needs within a relatively short period of time. For example, they wanted to have more space *“for playing, resting, and studying”* (Ethan, P2, TA), less scolding from teachers (Brandon, P3, WFA), or higher wages for their family (Queenie, P3, N-nothings).

*Having high extrinsic and intrinsic aspirations.* High aspirations refer to clear desires to set relatively greater goals for future achievements. The children who had higher aspirations showed a variety of hopes for the future. These aspirations can be seen as extrinsic, including desires for better lives and good occupations, as well as intrinsic, such as pursuits of moral goodness.

First, a group of children aspired to get better future lives in terms of material living. They shared similar hopes that they could get good education, good jobs with high earnings, and a big house, so they can treat their parents well as their parents gave their best to them.

*I hope I can study in a good university and get a good job. That way I can earn money and live in a big house.*

(Flora, S1, CSSA)

*I hope I can get a job after graduation and earn a lot of money for my family.*

(Isabel, P6, new immigrant)

*I want to let my parents live in a better and bigger house …and to have lots of good stuff.*

(Bridget, P4, WFA)

Second, some children, irrespective of their financial assistance or socioeconomic backgrounds, aspired for a variety of occupations, such as becoming an artist, engineer, scientist, teacher, E-sport player, medical doctor, and physical education teacher, which reflected their naturally inclined interests or fantasies. A male participant said that he wanted to be a scientist: *“…to invent a cleaning robot to sweep the floor. …I am too lazy to do the sweeping. The robot can help clean our house.”* (Oscar, P3, new immigrant). The children were also able to think in a sophisticated way by turning their interests or altruistic intentions into career options. A child expressed why he would like to become an E-sport player, and another expressed why he would like to become a doctor.

*I like playing games. I want my occupation to be game-related. …I hope I can make use of my hobby to earn money. …I am looking for an E-sport university. …My mom said the required scores are high and I have to first enter …a better school in Hong Kong.*

(Olivia, P5, TA)

*I want to be a doctor …to save people’s lives …because of the pandemic. …I could help a lot of people from dying and the scale of the pandemic would be smaller.*

(Patricia, P3, N-nothings)

Third, some aspirations were related to pursuits of moral goodness. Some children aspired to be morally virtuous and to contribute to society. For example, they said they would like to be honest (Ethan, P2, TA), to be helpful (Frank, P5, TA), or to be environmentally responsible (Ian, P3, new immigrant).

*Some people are quite poor …and we can help them …like donating some money to help them.*

(Hannah, P6, CSSA)

*I want there to be less rubbish in the world. There is too much rubbish in the landfill. …I want the rubbish to go away!*

(Ian, P3, new immigrant)

Another aspiration in relation to moral goodness is filial piety or intergenerational reciprocity, as shown by their desires to improve their parents’ future living. In Chinese culture, filial piety is a moral code that maintains intergenerational relationships or obligations [63]. Offspring are expected to reciprocate the care and provision their parents give them. The children also demonstrated their hopes to give their best to their parents in material terms.

A summary of all major themes is shown in Table 2.

## 5. Discussion

The results of this study have addressed two research questions regarding how poverty affects children’s lives and how children cope with the effects of poverty regarding different socioeconomic and environmental aspects including social life, healthcare, childhood possessions, etc.

### 5.1. The Effects of Poverty on Children’s Lives

Responding to the first research question, the findings showed how poverty affected children’s lives in three aspects. First, the economic and material deprivation aspect included basic needs (food, housing, clothing, and health care), childhood possessions (toys), and schooling resources. The children appeared to be food secure and free from hunger as they typically consumed three meals a day and felt they had balanced diets. Despite having sufficient food to eat, not all children acquired nutrients that were balanced enough. They also may not have enjoyed food choices that are part of social or cultural norms, such as dining out and consuming seafood. However, the children generally did not desire more or specific foods. Housing is a big problem for low-income children. They showed that they had limited living space and minimal room at home. What they demanded was modest, as they only asked for more personal space and basic home items. The children said that they had enough outfits, but this did not necessarily mean clothing was sufficient in terms of quality, as some of them had many unfitting clothes. School uniforms were adequate as they commonly had two interchangeable sets. School uniforms were also helpful in lessening the need for normal clothing during school days. Some children could see that other children had better clothing than theirs, while some children were not particularly bothered by comparisons with other children. The children felt that they were healthy. They tended to utilize informal health care, such as over-the-counter medicine and layman methods, though some children used formal, highly subsidized public health care. Formal private health care was less relied upon simply because of the lack of means.

As for childhood possessions, while some children said that they had a good quantity of traditional toys, many children stated that they did not play with toys as they felt they were too old for toys or that there was not enough space for toys. However, most of the children were fond of modern toys, referring to video games and smartphones. Some children realized that they lacked good game systems or smartphones, while some were indifferent to comparisons with others.

Regarding non-information technology schooling resources, many children wanted their own study desk and bookshelf. Regarding information technology resources, a lack of printers was the major concern. Children who did not have printers said that it bothered them as they had to seek help from their schools or NGO staff to print, or they had to copy by hand. Gadgets such as earphones were said to be important for online classes, especially in a small living space. With the donation of used digital devices by NGOs, the children were able to access online classes during the pandemic.

Another aspect of their lives that children shared about their experiences was social relationships and social participation, including peer relationships, family relationships, and extra-curricular activities. The school was shown to be an important context for them to socialize with friends. However, they seldom invited their friends to their homes because of limited living space and unfavorable home environments. They also had limited birthday party experience, including inviting friends for parties or going to their friends’ parties, as their limited home space or the need to buy gifts were disincentives for doing so. Birthday parties may also have been uncommon among low-income children as some children said their friends did not hold birthday parties. Most of the children enjoyed their family relationships, but long parental work hours reduced their interactions with parents. Despite some children having less family activities, most of them enjoyed brief leisure activities with their families, and for those who had family connections in mainland China, they enjoyed cross-border family trips during long holidays before the pandemic. With the support of NGOs, schools, and churches, the children were generally active in free or inexpensive extra-curricular activities. They said that cost was a major consideration for their decisions on participation. The pandemic was found to have reduced their participation in activities.

The third aspect of children’s lives was psychological and emotional wellbeing. It was revealed that their emotional wellbeing could be affected by their perceived family stability, in terms of relationship and financial conditions. They felt satisfied when their families were perceived as stable but felt worried when they were perceived as unstable. In regard to feelings about school lives, the children were mostly satisfied as they enjoyed their relationships with their peers and teachers. Moreover, the children had diverse aspirations, including low, moderate, and high levels of aspirations. Some with lower aspirations did not think much about their future. Others held moderate aspirations, such as more space or increased wages for families, and some held high extrinsic and intrinsic aspirations. High extrinsic aspirations included better future lives and good occupations, while high intrinsic aspirations referred to pursuits of moral goodness, which included reciprocal filial piety.

Taken together, as the children did not suffer from hunger, homelessness, a lack of clothing, a lack of health care, or a lack of education, their basic material needs seemed to be met. However, when looking beyond poverty, the children experienced a range of deprivations in the sense that they fell short of the resources that are commanded by average people and they could not fully participate in the relationships and activities that are normal to other people. An example is that despite the fact that children were sufficiently fed, the quality of their dietary intake may not have been adequate, and it was difficult for them to participate in common diet practices enjoyed by other people. The children were also disadvantaged in terms of social relationships and social participation. Long working hours for their parents, undesirable home environments, and limited income are factors that could undermine family interactions and opportunities to socialize with peers. It is also seen that even young children’s emotional wellbeing could be negatively affected by perceived family financial instability. Though the children might be deprived in their day to day living, they revealed a richness of their aspirations for the future. Overall, there was not an obvious pattern showing differences in the effects of poverty on children with different financial assistance or socioeconomic backgrounds.

In general, the findings of this study share similarities to existing literature, but they also show variations or unique aspects. For instance, it is similar to other studies in that the children are generally disadvantaged in different types of material deprivations, such as food insecurity [34,35] and overcrowded housing [40,43]. However, for the children in this study, limited living space stands out as a more severe problem that is urgent and more difficult to cope with. Certain material items they need for learning and daily living are distinct from the lists of child necessities in other studies [18,64]. Printers and small home appliances, such as ovens, washers, and vacuum cleaners, are seldom identified as essential in those studies. In addition, clothing was relatively less expressed as a concern in this study. In terms of social relationships, our findings are similar to other studies in that the children experienced barriers to peer relationships, as they seldom invited friends to their homes due to poor home conditions [40,43]. However, in this study, bullying and difficulty fitting in with peers due to clothing were less reported as an issue. Similar to the literature, the children in this study showed worries about their family’s financial situations [37,50] and they also exhibited diverse aspirations [39]. However, in regard to aspirations, more children in this study tended to hold relatively higher aspirations with a variety of goals. In particular, the desires for reciprocal filial piety appear to be more culturally unique for the children in this study.

### 5.2. The Coping Repertoire of the Children

As a response to the second research question, the results showed that children had various strategies to cope with their poverty experiences across different aspects of their lives. The various coping strategies included small spending savvy tactics, parental buffering, compensation, and mental coping.

#### 5.2.1. Small Spending Savvy Tactics

While the children may not have known the family budget details well, as it was their parents who managed and balanced the budget, they have been involved in small spending savvy tactics to cope with deprivation in their lives. One of the tactics employed to reduce living costs was getting the best deals. Examples were shopping in the wet market near its closing time, when many good deals were offered, and sharing fast food set meals with family members when dining out. Another tactic was taking pre-emptive moves to avoid unnecessary expenses, such as doing physical exercise to stay healthy in order to prevent medical expenses. There was also a common tactic of restraining spending by curbing one’s desires for material possessions to cut unnecessary buying. Many children seemed to have limited their longings for clothing, toys, or other items with justifications. Some children also reasoned that needs and wants were different and money should be spent on what was needed but not what was wanted.

These spending savvy tactics are similar to “getting by”, one of the four types of agency related to poverty [65]. Getting by refers to the little things that people do to cope with everyday situations, such as prioritizing daily expenditure and juggling resources [65,66]. Some studies of child poverty also found examples of what children do to get by, such as saving pocket money, taking advantage of ad hoc opportunities to earn income, and not complaining to parents about a lack of money [40,46].

#### 5.2.2. Parental Buffering

Parents could serve to moderate the impact of material deprivation on their children. As seen in the study, some parents tried to fulfil their children’s physical needs by sacrificing their own. For example, a mother would wear clothes and shoes outgrown by her child and cut her own clothing expenses in order to spend on her child. Parents would use over-the-counter prescriptions when feeling sick to save money while the children would visit the medical doctor. Parents would also try different ways to fulfil their children’s wishes that were normally unaffordable to them, such as a father bringing home seafood leftovers from a restaurant where he worked, as seafood was a relatively pricey food. These parental efforts may protect children from the chronic stress of living in impoverished and unhealthy conditions, which may result in poor physical, behavioral, and socioemotional development [67].

#### 5.2.3. Compensation

Compensation, in this study, refers to things or acts that help to offset or reduce the undesirable impacts of deprivation. It may also be considered as an everyday coping mechanism to get by [65]. There are different examples of compensation using alternatives to substitute for lacking items. Some children considered mobile phones to be replacements for video game consoles. To compensate for the small and noisy home environment, using a pair of earphones to listen to music or other social media could provide a little haven not only for a physical but also a psychological sense of space.

Travelling trips to mainland China can also be a compensation for the limited accommodation space and opportunities for family activities. As the majority of children lived in cramped living units without personal spaces, a short trip to mainland China could be a respite from the undesirable living conditions, physically or psychologically. The children loved to go on these trips as they could enjoy spacious indoor and outdoor environments while having fun with family and enjoying inexpensive food. The living expenses of the hometowns they went to were often very affordable. Moreover, holiday travel to other countries is a middle-class leisure in Hong Kong. While these children lacked the resources for overseas travelling, the trips across the border could have served as the best affordable substitutions for participating in a mainstream activity enjoyed by other more well-off children.

#### 5.2.4. Mental Coping

Making downward comparisons and positive thinking could be mental coping strategies for children. Some of the children tended to employ downward comparison to evaluate their circumstances by seeing that there were other people who were less fortunate, such as rough sleepers. To some children, this may have served as a means of self-protection for their self-esteem by fending off intimidating comparisons. Positive thinking could also have helped some children cope with the effects of poverty. Having a positive appraisal of their everyday situation was also a possible way to get by [65].

### 5.3. Issues on Improving Children’s Life Conditions

Based on the findings, several issues relevant to improving children’s life conditions are worthy of attention for policy measures and further research. They include food security issues, space and compensation, COVID-19 and poverty, protective resources for the children, and capitalizing on the aspirations of children.

#### 5.3.1. Food Security Issues

The children were food secure if food security was perceived as roughly having enough food to eat. However, they were not if the widely adopted definition of food security was referred to, which states that people, at all times, have physical, social, and economic access to sufficient, safe, and nutritious food to meet their dietary needs and food preferences for an active and healthy life [68]. Food security, therefore, contains four components: availability (physical availability of food), accessibility (having resources to obtain food), utilization (food accessed is of good quality), and stability (adequate availability, access, and utilization at all times) [69]. Thus, the children were food insecure in terms of accessibility, utilization, and stability, since some children had to rely on NGOs for their food supply from time to time because of limited resources to obtain food, and they were not able to obtain food that was balanced in terms of dietary intake. The extent to which food is insecure among children in poverty has yet to be understood by further comprehensive investigation into food security issues, and this could provide the necessary data to inform appropriate interventions to improve the food and nutrition conditions of children.

#### 5.3.2. Space and Compensation

Space is a unique issue in Hong Kong, especially in comparison with Western nations or other developed economies. Hong Kong is the fourth most densely populated territory in the world [70], and for the 11th year in a row, it remained the least affordable city in the world in terms of housing prices [71]. Limited living space is a commonly felt problem for children in poverty. While long-term housing policies will be required to solve the fundamental housing problems, some shorter-term measures can be promoted to improve the life conditions of children in terms of access to space.

One of the possible ways is to create more open areas and public facilities for fun in their proximal vicinities, especially in public housing estates and low-income neighborhoods. This would allow easy trips for children’s space enjoyment and a means to offset the sense of exclusion due to a lack of resources that are more available to other children. The need for good urban design and planning with attention given to these children is underscored.

Travelling trips, cross-border or local, could also improve children’s lives by compensating for space deprivation and increasing a sense of social inclusion. With its proximity to mainland China, the unique geographical location of Hong Kong offers a good option for this purpose. When the pandemic is under control and the border reopens, travelling trips to mainland China should be promoted for the children. Local trips within Hong Kong or to outlying islands have similar values. It is strongly recommended that the government should subsidize NGOs to organize more of both types of trips. To ease possible barriers for the children to fully enjoy space or local trips, certain facilitating resources, including travel subsidies and apparel and footwear suitable for the trips, should be ensured. Although local trips have been organized in the past by NGOs and schools for purpose of social exposure, these trips do entail an added value of compensation for space deprivation.

#### 5.3.3. COVID-19 and Poverty

Despite this study not focusing on the impact of pandemic on the children, the findings have reflected that the effects of the pandemic and poverty together could pose additional challenges to children in poverty. The pandemic has made the importance of digital resources for children’s learning prominent and could potentially widen the digital divide for children of different socioeconomic groups. During the pandemic, it was found that not only two-thirds of school-aged children worldwide lacked internet access at home, but there were also socioeconomic inequalities in internet access within countries. It was estimated that 16% of children and young people whose families were among the poorest 20% in their countries had internet access at home as compared to 58% of those from the richest 20% of families in their countries [72]. It was fortunate that the children in this study were able to access digital devices, as some of them received help from NGOs. However, children’s lives in other aspects were more affected. For instance, school shutdowns or later partial school resumption had limited their interactions with peers. Social participation was largely hampered as extra-curricular activities were suspended. Family trips to mainland China became impossible. Although other children in better circumstances may have faced similar issues, their better home environment and better access to educational or recreational resources at home were likely to ease the negative impacts of the pandemic. More research could be conducted to further understand the disparities in experience among children of different socioeconomic levels. This may give insights into how to lessen the effects of the pandemic on children of different backgrounds.

#### 5.3.4. Protective Resources for Children

This study showed that the non-profit sector played a valuable role in protecting children from the impact of material deprivation in Hong Kong. The children have benefited from the assistance of NGOs in gaining access to food, clothing, digital devices, and social activities. Without their help, the situations of the children would be worse. In addition to acknowledging the efforts of NGOs, their role in improving children’s lives could be further strengthened. The government should devote more resources to the NGOs so that their capacities for meeting children’s needs can be enhanced to improve all facets of their lives.

Family relationships or parental buffering are shown to be important protective factors to shield the children from the negative impacts of poverty [73]. A possible approach is for NGOs to organize various family activities to support parents and to promote family interactions. This may also encourage the parents to be more familiar with and supportive to the work of NGOs, which in turn would facilitate their efforts in improving children’s lives.

#### 5.3.5. Capitalizing on Children’s Aspirations

**Figure 1:**
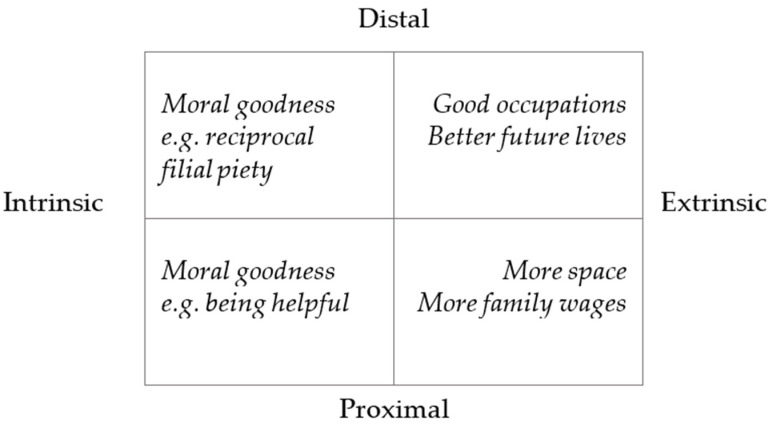
The complexities of children’s aspirations.

The children in this study showed a range of low to high aspirations for the future. The moderate and high aspirations of the children can generally be viewed in two dimensions: intrinsic or extrinsic nature, and short or long term. Extrinsic aspirations refer to aspirations that are concerned with getting out of poverty, while intrinsic aspirations refer to those that refer to becoming morally altruistic, such as being helpful, being filial or reciprocal, and contributing to society. Moreover, some aspirations, such as career aspirations, are long-term achievements, while other aspirations, such as helping people, can be achieved within a shorter period of time. The sophistications of children’s aspirations can be seen in Figure 1.

Children in poverty are shown to have agency and the capacity to aspire to a variety of goals. Instead of simply treating the children as victims or recipients of assistance, it would be meaningful to focus and capitalize on their existing aspirations for the further cultivation and conversion of these aspirations into reality. For example, given their desires to be virtuous and contributive, a possible approach could be the promotion of volunteering service among the children, whereas volunteering was rarely mentioned as part of their leisure time activities. Schemes of volunteering services organized by NGOs could not only increase the children’s social exposure but could also be platforms for nurturing the intrinsic motivations of the children and the pursuit of long-term goals.

## 6. Limitations and Future Research

This study has its own limitations. There is a sample selection issue. This study included more children participants who live near the poverty line and in two-parent families than those who live under the poverty line and in single-parent families. This implies that the study may not have reflected fully the lived experience of children who may fare even worse. This is also a possible reason why there is no obvious difference in the effects of poverty on children across different financial assistance or socioeconomic backgrounds. In addition, this study only purposively sampled child participants via the NGO network known to the research team. The NGO network is not extensive enough to sample children who are of diverse disadvantaged backgrounds, such as children with a disability or multiple disabilities, ethnic minority children, children whose parents having mental health challenge or criminal record, children living in disadvantaged neighborhoods, etc. We therefore need to be cautious about drawing any definite conclusions regarding the transferability of our research findings and analysis to many other different types of children in poverty, such as children in deep poverty, single-parent households, children with physical disabilities or mental health challenges, deprived communities, children with young parents, etc. Similar caution should also be taken when making policy recommendations based on the findings.

In future research, a more balanced sample that includes more children who live under poverty and who live in single-parent households should be considered. Inviting child participants from a broader spectrum of NGOs in many more districts over Hong Kong, which are committed to working with different types of children, can help address the sampling issue as well. Various issues, including food insecurity among poor children and disparities in the effects of the pandemic on children of different socioeconomic levels, can be further investigated in future studies.

## 7. Conclusions

This study revealed that children’s perceptions of their own lived experience in poverty or near poverty with regard to different aspects of their life domains, including livelihood, study, recreation, social participation, and diverse material aspects including but not limited to clothing, possessions of toys, dietary practice, use of healthcare resources, etc., and their approaches to coping with life characterized by relative deprivations in terms of both tangible and intangible aspects, have to be taken into thorough consideration. Children as young people are not passive but active agents in making sense of their own lived experience and social relationships which are situated within specific socioeconomic and environmental contexts. Children’s proximity and access to their own schools, neighborhoods, and NGOs as their zone of participation and empowerment, which can be leveraged as a means to further enhance their wellbeing and sense of belonging, has to be thoroughly addressed through further research studies in order to inform the design, delivery, and evaluation of quality services to children in or at the margin of poverty in developed economies in particular.

## Figures and Tables

**Table 1:** Background characteristics of the child participants.

Participant	Age	Gender	Education Level	Family Type	Status of Financial Assistance and Other Socioeconomic Backgrounds
1. Aaron	11	M	P5	Two parents	WFA
2. Amelia	10	F	P4	Single parent	CSSA
3. Bridget	11	F	P4	Two parents	WFA
4. Claire	10	F	P4	Single parent	CSSA
5. Daniella	11	F	P6	Two parents	WFA
6. Brandon	9	M	P3	Single parent	WFA
7. Eleanor	9	F	P3	Single parent	CSSA
8. Flora	11	F	S1	Single parent	CSSA
9. Giselle	8	F	P3	Two parents	CSSA
10. Hannah	14	F	P6	Two parents	CSSA
11. Isabel	14	F	P6	Two parents	New immigrant
12. Caleb	9	M	P3	Two parents	TA scheme
13. Derrick	13	M	S1	Two parents	TA scheme
14. Ethan	9	M	P2	Two parents	TA scheme
15. Frank	11	M	P5	Two parents	TA scheme
16. Gerald	9	M	P3	Two parents	New immigrant
17. Jenny	8	F	P3	Two parents	New immigrant
18. Hayden	12	M	S1	Two parents	TA scheme
19. Kate	10	F	P4	Two parents	TA scheme
20. Lucia	12	F	P5	Two parents	CSSA
21. Ian	8	M	P3	Two parents	New immigrant
22. Justin	11	M	P4	Two parents	TA scheme
23. Kyle	9	M	P3	Two parents	TA scheme
24. Leo	10	M	P5	Two parents	TA scheme
25. Max	12	M	P6	Two parents	TA scheme
26. Mia	9	F	P3	Two parents	TA scheme
27. Natalie	12	F	P6	Two parents	TA scheme
28. Nick	10	M	P5	Two parents	TA scheme
29. Oscar	8	M	P3	Two parents	New immigrant
30. Perry	11	M	P5	Two parents	TA scheme
31. Rex	9	M	P4	Two parents	TA scheme
32. Olivia	11	F	P5	Two parents	TA scheme
33. Sean	10	M	P4	Two parents	TA scheme
34. Patricia	8	F	P3	Two parents	N-nothings
35. Queenie	8	F	P3	Two parents *	N-nothings
36. Todd	11	M	P5	Two parents	TA scheme
37. Ruth	9	F	P3	Two parents	TA scheme
38. Vance	9	M	P3	Two parents	CSSA
39. Wallace	12	M	P5	Two parents	N-nothings
40. Zack	9	M	P3	Two parents	N-nothings

Note: * Father in Mainland China. CSSA = Comprehensive Social Security Assistance Scheme, WFA = Working Family Allowance Scheme, TA scheme = School Textbook Assistance with full grant, N-nothings = low-income households without government welfare, and new immigrants = immigration from mainland China within five years.

**Table 2:** A summary of major themes.

Aspects of Poverty Experience	Major Themes
1. Economic and material deprivation	
Basic needs Food	Being food secure without going hungry Being deprived of socially or culturally desirable food practices
Housing	Limited living space Minimal set up
Clothing	Sufficient in quantity but not necessarily in quality
Health care	Heavy reliance on informal health care
Childhood possession	Lack of popularity of traditional toys Fondness of modern toys
School resources	Lack of non-information technology resources Lack of a printer General accessibility of other digital devices
2. Social relationships and social participation	
Peer relationship	School as a major setting for friendship Limited opportunities to celebrate special occasions with peers
Family relationship	Family interactions depending on parental work From simple family leisure to cross-border trips
Extra-curricular activities	Generally high participation in free-of-charge extra-curricular activities
3. Psychological and emotional wellbeing	
Feelings about family circumstances	Emotional wellbeing affected by perceived family stability
Feelings about school lives	Mostly satisfied with school lives
Aspirations	Different levels of aspirations: low, moderate, high

## Data Availability

The data presented in this study are available from the corresponding author upon reasonable request.
